# An Experimental Study on the Frictional Behavior of Ultrathin Metal Sheets at Elevated Temperatures

**DOI:** 10.3390/ma17123009

**Published:** 2024-06-19

**Authors:** Yuhang Xia, Zeran Hou, Jinjun Tan, Wenyao Wang, Nan Guo, Junying Min

**Affiliations:** School of Mechanical Engineering, Tongji University, Shanghai 201804, China; xiayuhang007@163.com (Y.X.); 2330375@tongji.edu.cn (J.T.); wenyao_w@163.com (W.W.); 13guonan@tongji.edu.cn (N.G.)

**Keywords:** ultrathin metal sheets, high temperature, frictional behavior, dynamic measurement

## Abstract

Hot forming is an effective approach for improving the formability of ultrathin metal sheets, such as those made of stainless steel and pure titanium. However, the increased friction coefficient between the tool and the high-temperature metal sheet negatively affects material flow during hot forming, potentially resulting in severe local thinning or even cracking. This study explores the frictional behavior of 0.1 mm thick ferritic stainless steel (FSS) and commercially pure titanium (CP-Ti) sheets at elevated temperatures. A friction testing apparatus was developed to measure the friction coefficients of these metal sheets from room temperature (25 °C) up to 600 °C. The friction coefficient of the FSS sheet increased monotonically with temperature, whereas that of the CP-Ti sheet first increased and then decreased. Post-friction testing microscopic examination demonstrated that built-up edges formed on the surfaces of the friction blocks when rubbed against the stainless steel, contributing to the higher friction coefficients. This study provides a foundation for understanding frictional behavior during the hot forming of ultrathin metal sheets.

## 1. Introduction

Metallic components and miniature structures have been widely applied in various fields, including electronic devices [1], microelectromechanical systems [2], and fuel cells [3]. The forming of ultrathin metal sheets has become a highly efficient and cost-effective technology for mass-producing metallic microcomponents and structures [4]. However, conventional cold-forming processes are insufficient for fabricating ultrathin sheets [5,6]. Based on the fact that increasing the forming temperature can effectively reduce the strength of metallic materials and enhance their plasticity [7], the hot-forming process is better suited for manufacturing ultrathin metal sheets. Guo et al. demonstrated this by manufacturing ultrathin SS316L bipolar plates using hot stamping, which enhanced their dimensional accuracy and uniformity [8]. Similarly, Modanloo et al. proposed a warm stamping process to manufacture titanium bipolar plates that improved the channel depth and reduced the springback and thinning rates of the channel [9]. Zheng et al. conducted microbending experiments at different temperatures to investigate the effect of temperature on the springback behavior of pure titanium foil [10]. The findings of these studies demonstrated that increasing the forming temperature can effectively reduce the springback angle of the formed parts. Zhang et al. found that the fracture limits of ultrathin titanium sheets at 700 °C are higher than those at room temperature (RT), enabling the successful manufacture of crack-free titanium bipolar plates via hot stamping [11]. However, Narayan studied the stamping process of ferritic stainless steel (FSS) bipolar plates and observed that the friction between the sheet and die significantly increased at a forming temperature of 200 °C, leading to strain concentration and necking at the flow channel corners [12]. This issue was mitigated using lubricants, which prevented necking and enhanced the uniformity of the thickness distribution.

The friction coefficient, an intuitive parameter for characterizing the frictional behavior between friction pairs, also serves as a fundamental input value for determining the frictional force between contact pairs in finite element simulations. Therefore, accurately measuring the friction coefficient between contact pairs has become a central research area. Numerous test methods, such as stretch bending [13], tensile strip [14], probe [15], and sliding tests [16], are available for sheet-metal-forming friction experiments. However, high-temperature friction coefficient measurements are extremely challenging. Several researchers developed test systems to measure the friction coefficients of various materials and manufacturing processes at high temperatures using the aforementioned four typical friction coefficient test methods. Venema et al. constructed a hot-friction drawing tester using the sliding test method [17]. The test platform connects the heating furnace and sliding friction test device in series, allowing sliding friction experiments to be conducted on a 1.5 mm thick press hardening steel at 450–750 °C. Shisode et al. employed a rotating friction tester (RFT) to measure the friction coefficient of 0.7 mm thick zinc-coated steel sheets at different pressures [18]. The RFT consisted of a stationary punch and rotating sample holder, which could apply a maximum normal pressure of 60 MPa and a sliding speed of 100 m/s to the sample. Bastovansky et al. developed a strip test device suitable for ordinary tensile testing machines [19]. This equipment enables strip sliding friction tests to be conducted on sheet metal specimens up to 50 mm wide and 1 mm thick at a maximum temperature of 400 °C. The measured drawbead restraint force and holder drawing force were used as fundamental parameters to calculate the friction coefficient. In summary, the current research on the frictional behavior at the mesoscale is mostly conducted using macroscopic friction testing devices, which cannot accurately characterize the friction coefficient of ultrathin sheets (thickness of only 0.1 mm), especially in the absence of relevant reports on testing devices specifically designed for ultrathin sheets at high temperatures.

Therefore, understanding the frictional behavior of ultrathin metal sheets at elevated temperatures is essential. A specialized testing apparatus was developed in this study to measure the friction coefficients of ultrathin (0.1 mm thick) FSS and commercially pure titanium (CP-Ti) at elevated temperatures. The post-friction testing surface morphologies were examined using three-dimensional (3D) topography scanning to reveal the underlying mechanisms of high-temperature friction in ultrathin metal sheets.

## 2. Materials and Methods

### 2.1. Materials

This study employed 0.1 mm thick 446 FSS and CP-Ti sheets, owing to their increased forming limits via hot-forming, and their potential applications in fabricating bipolar plates [8,11,20]. The CP-Ti was annealed after cold rolling to obtain a titanium substrate with an α-phase structure. Table 1 and Table 2 list the chemical compositions (wt. %) of the 0.1 mm thick FSS and CP-Ti sheets, respectively. The chemical compositions were measured using X-ray fluorescence (XRF) analysis.

### 2.2. Friction Tests 

#### 2.2.1. Testing Apparatus

**Figure 1:**
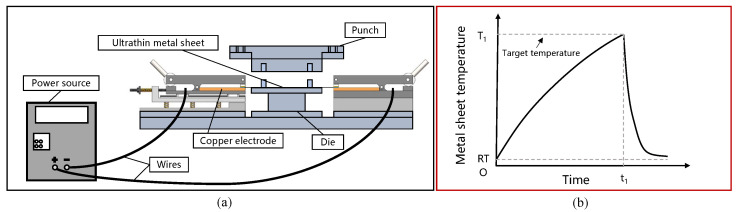
(**a**) Schematic diagram of the hot stamping platform developed by Guo et al. [8]. (**b**) Temperature vs. time curve of an ultrathin metal sheet during the hot stamping process.

**Figure 2:**
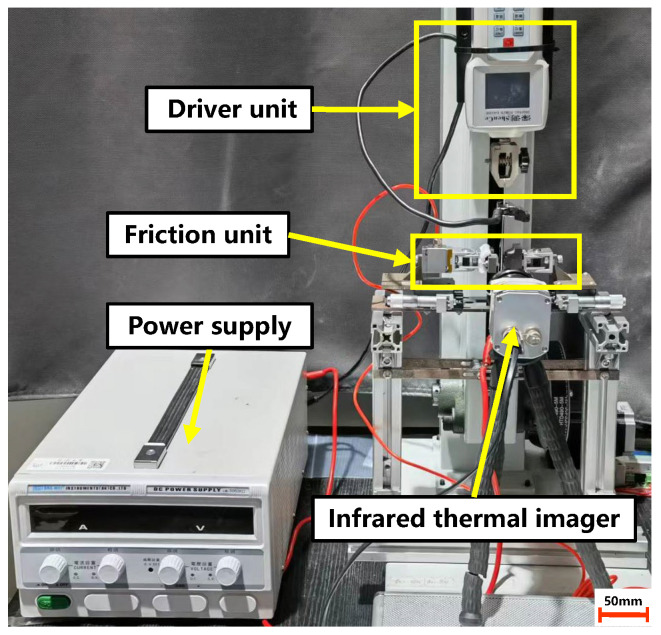
Testing apparatus developed for this study.

Resistance heating is considered a promising method for hot stamping, owing to its ability to rapidly heat ultrathin metal sheets and obtain a uniform temperature field [21]. Guo et al. developed a laboratory-scale hot stamping platform for the high-temperature formation of ultrathin metal bipolar plates using resistance heating [8]. Figure 1a,b show, respectively, a schematic diagram of the hot stamping platform, containing an on-site resistance heating system, and a temperature–time curve of an ultrathin metal sheet during the hot stamping process. Hence, a homemade friction testing apparatus, which simulates the resistance heating high-temperature forming process, was developed in this study to measure the friction coefficients of ultrathin metal sheets at elevated temperatures. As illustrated in Figure 2, our homemade friction testing apparatus comprises a driver unit, DC power supply, friction unit, and infrared thermal imager. 

The primary principle of this testing apparatus is that the DC power supply unit outputs current through two electrodes to heat the ultrathin metal sheet. The specimen temperature is measured using an infrared thermal imager featuring a minimum thermal sensitivity (NETD) of 0.08 K and an accuracy of ±0.2 °C. Once the target temperature is reached, the friction unit is brought into contact with the sample, and a certain pressure is applied. The pressure is measured using a force sensor with a range of 0−100 N and an accuracy of ±0.01 N, which is installed within the friction unit. Subsequently, the driver unit pulls the specimen, allowing sliding friction between the specimen and the friction unit. The driver unit, powered by a motor and capable of maintaining even stretching at speeds ranging from 3–−300 mm/min, pulls the specimen against the friction block while a force sensor with an accuracy of ±0.01 N records tension in real time. The DC power supply unit, capable of delivering a maximum current of 60 A and voltage of 50 V, is linked to the clamping section of the specimen via a wire. This setup allows for precise control of the maximum heating temperature of the sheet, up to 1050 °C. 

**Figure 3:**
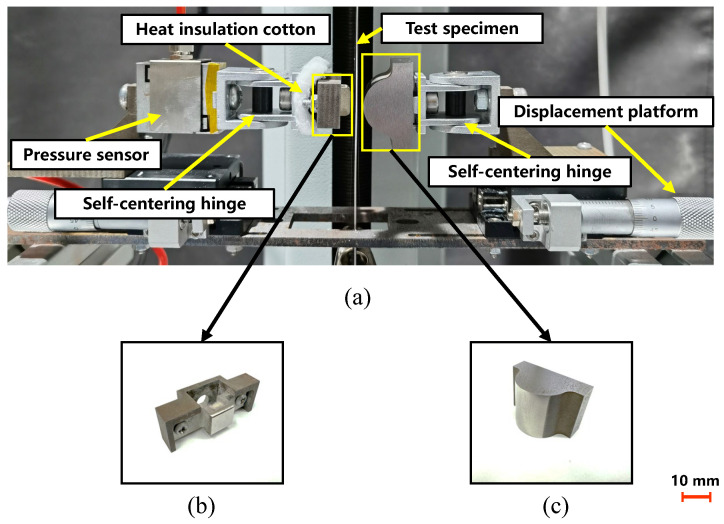
(**a**) Friction unit, (**b**) flat friction block, and (**c**) semi-cylindrical friction block.

Figure 3 shows the friction unit used in this study, which consists of two friction blocks, one flat and one semi-cylindrical, designed to counteract non-parallelism arising from installation discrepancies. The design of the flat friction block, shown in Figure 3a, effectively reduces heat loss during the electric heating of the specimen and allows it to rapidly reach the target temperature during high-temperature friction. The friction block was fabricated using Cr12MoV (HRC53-58), a common tool material used in hot stamping. Self-centering hinge joints were used in this study to ensure that the semi-cylindrical axis was parallel to the friction plane of the flat friction block. The following two measures were implemented to mitigate the effect of temperature on the accuracy of the pressure sensor: using a self-centering hinge between the block and sensor to extend the heat transfer path and enhance heat dissipation; and placing insulating cotton between the block and the hinge. Preliminary tests validated that this setup effectively minimized sensor output fluctuations caused by temperature increases. A displacement platform was used to adjust the position of the friction block relative to the specimen, and to control the pressure exerted by the friction block on the specimen. Moreover, the friction block was polished using a 6 μm diamond agent to match the surface roughness of the hot stamping tool (Ra 0.3). Surface roughness measurements of the friction unit were conducted using a 3D contour measurement instrument (KEYENCE, VR-5000, Osaka, Japan), featuring a display resolution of 0.1 μm and an accuracy of 0.5 μm. The final surface roughness of the flat block was determined to be Ra = 0.29, whereas that of the semi-cylindrical block was Ra = 0.24.

#### 2.2.2. Testing Specimen

**Figure 4:**
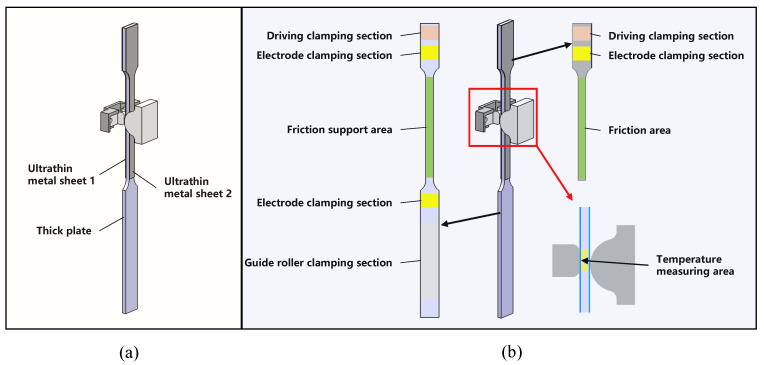
(**a**) Sandwich structure and (**b**) role of each part of the ultrathin metal sheets and thick plate.

The electric current-heated 0.1 mm thick metal sheets cooled rapidly when in contact with the friction block, which made it challenging to reach the target temperature and measure the temperature in the friction region. Therefore, a novel sandwich structure was designed in this study, as shown in Figure 4a. The structure consisted of two ultrathin sheets sandwiched between an intermediate 1 mm thick plate. The thick plate was heated using a current to prevent excessive cooling of the specimen after contact with the friction block. The three plates in the sandwich structure were stacked directly and secured tightly by the clamps of the driver unit, which ensured maximum heat transfer from the thick plate to the ultrathin sheets on both sides, and provided heat preservation during the friction process. A reflective paint of known emissivity was applied to the sides of the thick plate, allowing its temperature to be measured in real time using an infrared thermal imager, as depicted in Figure 4b. The thick plate was designed with a “dog bone” shape, with a narrow middle and wide end to ensure uniform heating of the friction section. The ultrathin metal sheet retained only the clamping and friction sections. Figure 4b defines the roles of each part of the thick plate and ultrathin metal sheet.

**Figure 5:**
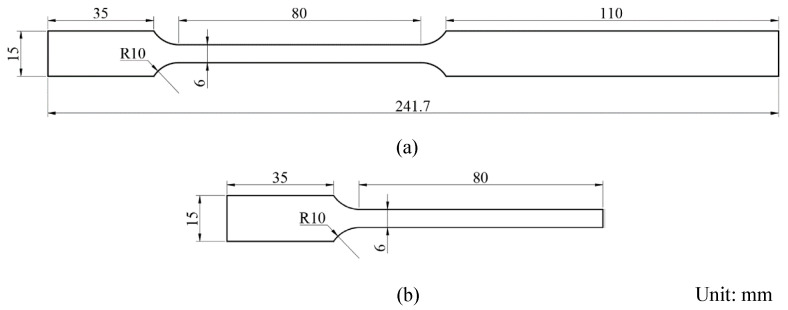
Geometric dimensions of (**a**) the intermediate thick plate and (**b**) the ultrathin metal sheets.

Figure 5a,b show the specific geometries of the thick intermediate plate and ultrathin metal sheets, respectively. The ultrathin metal sheets and intermediate thick plates were processed using low-speed wire cutting. Before testing, the surfaces of the ultrathin metal sheets were cleaned with alcohol to remove surface dirt. The edges of the sheets were polished using sandpaper to prevent the wire-cutting-generated burrs from affecting the experimental results. Additionally, the length of the ultrathin sheet was perpendicular to the rolling direction of the sheet during processing to prevent rolling scratches from interfering with the friction test. The friction support section of the intermediate thick plate was progressively polished using sandpaper, culminating in a final polish with a 6 μm polishing agent. This process aimed to achieve a surface roughness level lower than that of the ultrathin sheet, which was Ra 0.4.

#### 2.2.3. Testing Procedure

The entire testing procedure consisted of the following three phases based on the temperature history: (1) the specimen was heated, and its temperature increased; (2) the temperature of the specimen decreased after getting in contact with the friction block; and (3) friction occurred between the specimen and friction block. In phase (1), the sandwich structure was heated using a constant-current method, and the temperature was monitored using an infrared thermal imager. Once the temperature of the specimen stabilized, the friction block moving platform was rotated to bring the two friction blocks into contact with the specimen and apply pressure, thus entering phase (2). During phase (2), the knob on the friction block moving platform was adjusted to reach the set pressure value. In this work, the normalized pressure used was calculated as follows:

(1) $$N^{*}=\frac{N}{YS_{n}}$$

where $N^{*}$ is the normalized pressure, N is the pressure applied in the experiment, Y is the yield strength of the material, and $S_{n}$ is the nominal contact area determined from finite element simulations. Because the mechanical properties of the ultrathin metal sheets vary with temperature, applying the same pressure regardless of temperature would yield significantly different levels of material deformation. Consequently, different pressure values were set for FSS and CP-Ti at different temperatures. For FSS, a normalized pressure of 0.01 was selected, resulting in actual pressures of 5, 3.9, 3.2, and 3.2 N at RT, 200, 400, and 600 °C, respectively. For CP-Ti, a normalized pressure of 0.05 was selected, corresponding to actual pressures of 14.8, 11.3, 7.2, and 4.2 N at, RT, 200, 400, and 600 °C, respectively. Subsequently, the friction drive device was activated to initiate phase (3). The test was repeated thrice under the same conditions to record the pressure and tensile force and observe friction conditions on the specimen surface.

## 3. Results and Discussion

### 3.1. Validation of the Testing Apparatus

**Figure 6:**
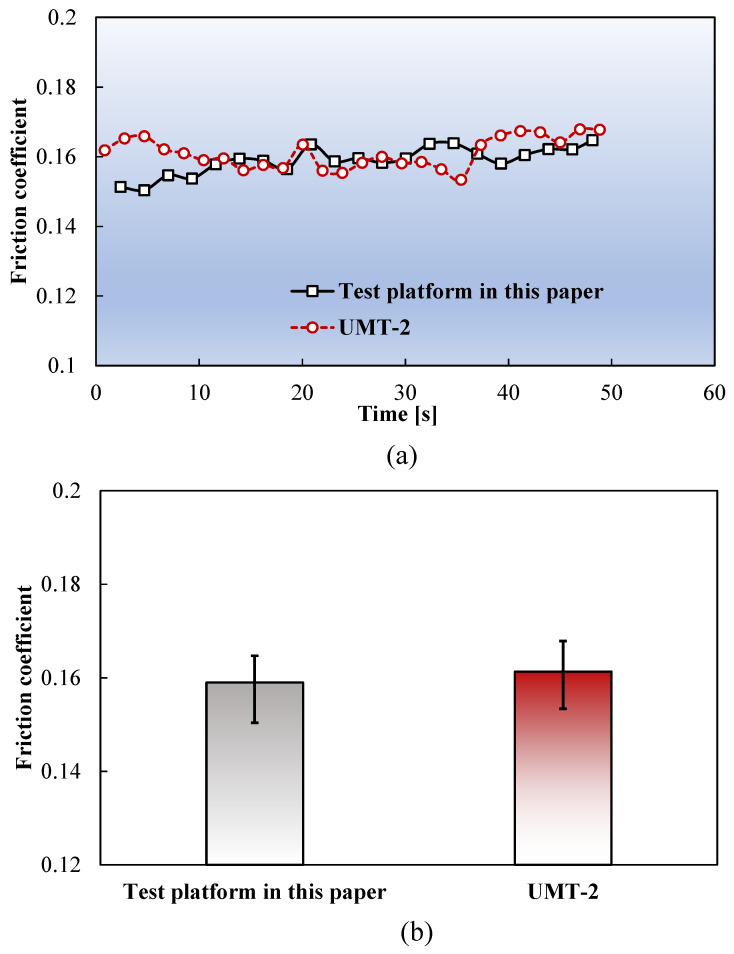
(**a**) Friction coefficient as a function of time and (**b**) mean friction coefficient of the two devices.

A control experiment was conducted using a tribometer (UMT-2, Bruker, Billerica, MA, USA) to validate the accuracy of the testing apparatus developed in this study. As the UMT-2 tribometer could not fulfill the high-temperature testing requirements of ultrathin metal sheets, the comparison was performed at RT using a normal pressure of 10 N and a friction speed of 3 mm/min. Figure 6 compares the test results. The results of the friction coefficients measured by the apparatus shown in Figure 2 and that measured using the UMT-2 tribometer demonstrate good agreement, validating the effectiveness of the friction apparatus developed in this study.

**Figure 7:**
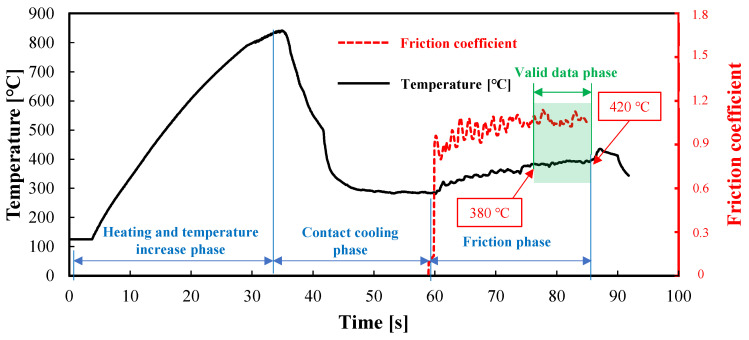
Temperature history and friction coefficient curves during the experimental process.

The testing apparatus was used to measure the high-temperature friction coefficient, and temperature and friction coefficients as a function of time curves were obtained, as shown in Figure 7. This process serves as an example for conducting friction coefficient measurements at 400 °C. The temperature history was recorded using an infrared thermal imager. Moreover, the driver unit and pressure sensors output time–force data during the experiment. Considering that each force sensor operated independently, synchronizing the data over time to match the tension and pressure was necessary. The friction coefficient was calculated using Equation (2), as follows:

(2) $$\mu =\frac{F}{2N’},$$

where F represents the friction data obtained by the tension sensor and N’ represents the normal pressure data obtained by the pressure sensor. Because the specimen interacted with the left and right friction blocks, the calculated friction coefficient was divided by two to obtain the actual value. The actual temperature in the contact area between the sheets and friction block remained dynamic. Therefore, this paper proposes a dynamic measurement and data processing method for determining the friction coefficient. Since the lower end of the specimen remained uncooled because it was not in contact with the friction block, its temperature was significantly higher than that of the friction region during the friction phase (phase (3)). Therefore, as the tension device stretched the specimen upwards, the temperature in the friction region gradually increased. When the temperature was within 10% of the expected value (for example, the expected value is 400 °C; therefore, the measured temperature range is 380–420 °C), the friction force, positive pressure, and moving distance were recorded. The temperature values shown in Figure 7 are the average temperatures of the temperature measuring area depicted in Figure 4. The average friction coefficient within this range was recorded as the friction coefficient of the sheet metal at the desired temperature. Finally, the friction coefficient time curves of the ultrathin metal sheets at elevated temperatures were obtained.

### 3.2. Effect of Temperature on the Friction Coefficient

**Figure 8:**
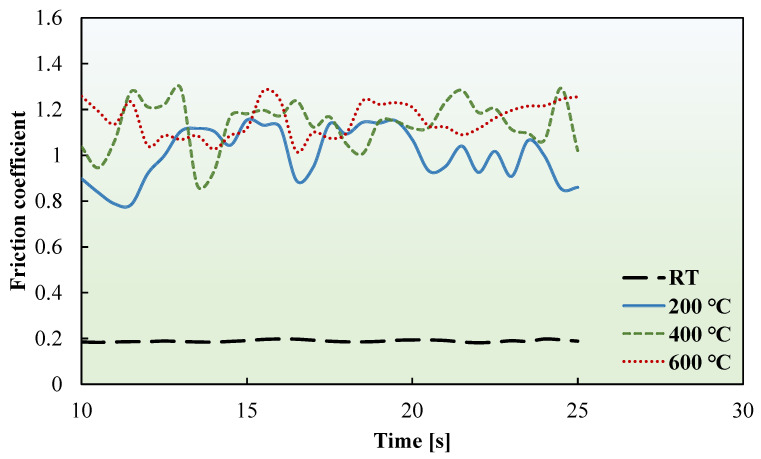
Friction coefficient curves of FSS sheet at RT, 200, 400, and 600 °C.

**Figure 9:**
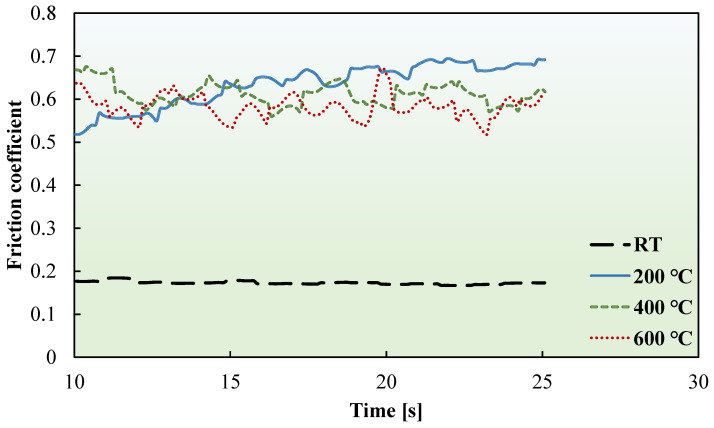
Friction coefficient curves of the CP-Ti sheet at RT, 200, 400, and 600 °C.

The friction coefficients of the ultrathin FSS and CP-Ti sheets were measured at RT, 200, 400, and 600 °C. Figure 8 and Figure 9 present the friction coefficient curves of the FSS and CP-Ti sheets, respectively. These curves illustrate the dynamic measurement process of the ultrathin metal sheets at various temperatures.

**Figure 10:**
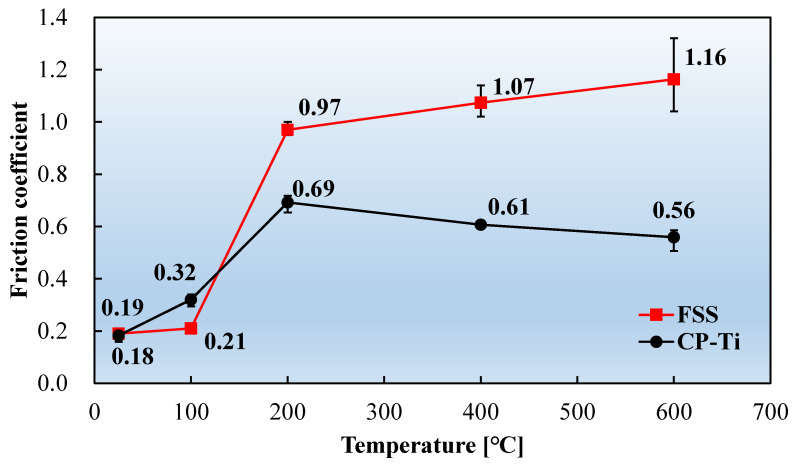
Friction coefficients of the FSS and CP-Ti sheets as a function of the temperature.

Figure 10 compares the friction coefficients of the two materials as a function of the temperature. Notably, the friction coefficient significantly varied in the temperature range from RT to 200 °C. Therefore, supplementary experiments were conducted on the two materials at 100 °C. The friction coefficients of the two materials exhibited slightly different trends with increasing temperature. Friction coefficients of both FSS and CP-Ti rapidly increased with temperature, up to 200 °C, followed by a gradual increase for the FSS material and a slight decrease for the CP-Ti. The friction coefficients of the FSS and CP-Ti sheets at RT were low, being 0.19 and 0.18, respectively. When the temperature was increased to 200 °C, the corresponding friction coefficients increased rapidly to 0.97 and 0.69, representing increases of 411% and 278%, respectively. The supplementary experiments revealed that the respective friction coefficients of FSS and CP-Ti at 100 °C were 0.21, which is not significantly different from that at RT, and 0.32, which is considerably higher than that at RT. The average friction coefficient between the FSS sheet and the friction block increased slowly at temperatures > 200 °C, while the sensitivity of the friction coefficient to temperature decreased. The average friction coefficient between the CP-Ti sheet and the friction block at temperatures > 200 °C was less sensitive to temperature than that of FSS. The average friction coefficient of the CP-Ti sheet gradually decreased from 0.69 at 200 °C to 0.56 at 600 °C, representing a reduction of 19.2%.

### 3.3. Mechanisms of High-Temperature Friction in Ultrathin Metal Sheets

The experimental results indicate that the friction coefficients of FSS and CP-Ti increased rapidly between RT and 200 °C, and exhibited opposing trends with temperature variation. The KEYENCE VR-5000 3D contour measurement instrument was used to examine the surface of the specimen and friction block after friction to clarify the friction mechanism. 

**Figure 11:**
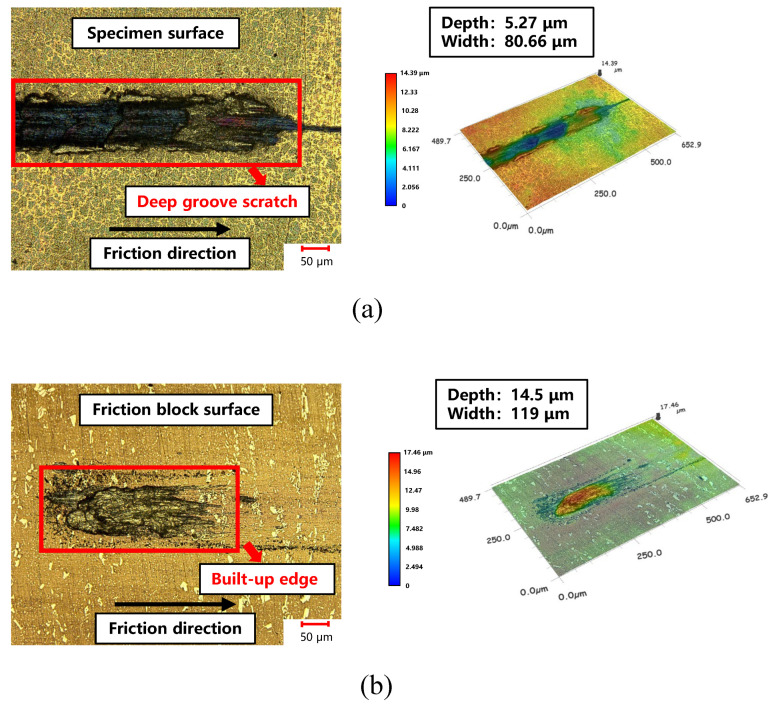
(**a**) Deep groove scratch on the specimen surface. (**b**) Built-up edge on the friction block surface.

Figure 11a shows the friction morphology and scratch size of the FSS sheets at 400 °C. The maximum friction coefficient between the specimen and the friction block during the test was 1.1, with the scratch depth and width measured at 5.27 μm and 80.66 μm, respectively. Notably, these deep groove scratches did not cause scratches on the other contact surfaces of the specimen in contact with the friction block, indicating their significant influence on the frictional behavior of ultrathin metal sheets at high temperatures. The surface of the friction block in contact with the specimen was examined, as shown in Figure 11b. Rough features were generated on the surface of the friction block. Furthermore, the scratches on the specimen surface deepened, suggesting that these lumps were built-up edges formed during friction between the test specimen and the friction block.

**Figure 12:**
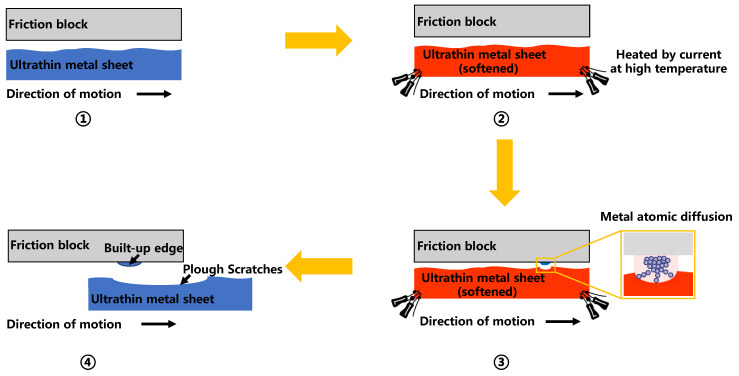
Formation mechanism of the built-up edge.

Frictional force primarily arises from plowing and adhesive forces [22,23,24]. Venema [25] and Wang [26] suggested that the atoms in both contacting materials are highly activated at high temperatures, facilitating mutual diffusion at the material surfaces, and leading to the formation of strong chemical bonds. Concurrently, the material softens and becomes more prone to deformation at higher temperatures. These two factors contributed to the formation of a built-up edge that adhered to the surface of the friction block. The built-up edge continually peeled off during the interaction until peeling and bonding reached equilibrium, resulting in a stable friction coefficient. Figure 12 illustrates the formation mechanism of the built-up edge.

**Figure 13:**
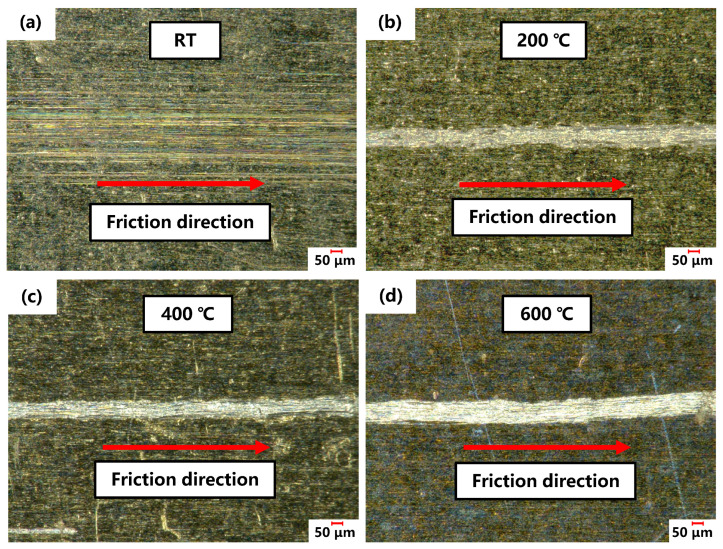
Micrographs of the scratches on the FSS specimen surface at (**a**) RT, (**b**) 200, (**c**) 400, and (**d**) 600 °C.

**Figure 14:**
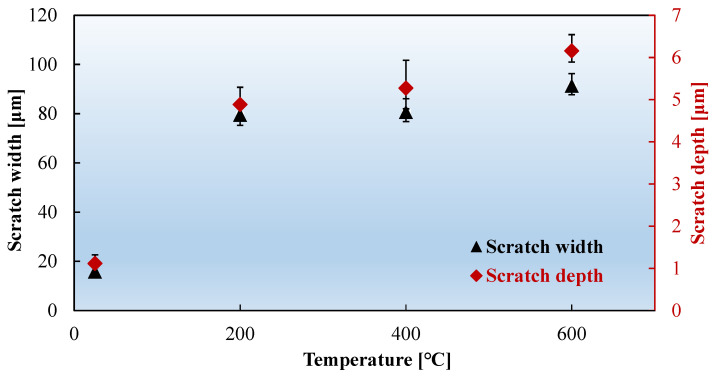
Scratch depths and widths on the FSS sheet at different temperatures.

The surfaces of the friction specimens were examined using a super depth-of-field microscope. Figure 13 shows the scratches on the surface of the FSS sheet after friction testing at different temperatures. The scratch dimensions were measured as shown in Figure 14. The scratches at RT were shallow, sparse, and evenly spread across the surface, offering little resistance to motion, and thus leading to a low friction coefficient. However, the appearance of deep and wide scratches at 200 °C suggests significant wear, indicating the formation of a built-up edge on the surface of the friction block. This edge impedes movement, causing the friction coefficient to spike from 0.29 at RT to approximately 1.04. Notably, the friction coefficient of FSS at 100 °C remained comparable to that at RT, with no significant increase. As previously discussed, the friction coefficient at high temperatures depends on material softening and atomic activation. The yield strengths of FSS at RT and 100 °C were 467 and 450 MPa, respectively. FSS did not undergo significant softening, resulting in no significant change in the friction coefficient. As the temperature increased further, the friction coefficient slowly increased and then stabilized, following a similar pattern to the scratch width and depth, attributed to the significant decrease in the yield strength of the sheet, and the gradual increase in the proportion of material softening, which offset the influence of the plowing effect of the built-up edge and stabilized the friction coefficient. Although some scholars have pointed out that an oxide layer forms on the surface of stainless steel under high-temperature conditions, affecting the friction coefficient [27,28], in reality, its impact is limited. The friction coefficient in sliding friction behavior caused by the oxide layer does not exceed 1. The experimental friction coefficients and the 3D scanning results of the specimen and friction block surfaces show that the friction is not merely a simple sliding friction; the primary influencing mechanism is the formation of a built-up edge.

**Figure 15:**
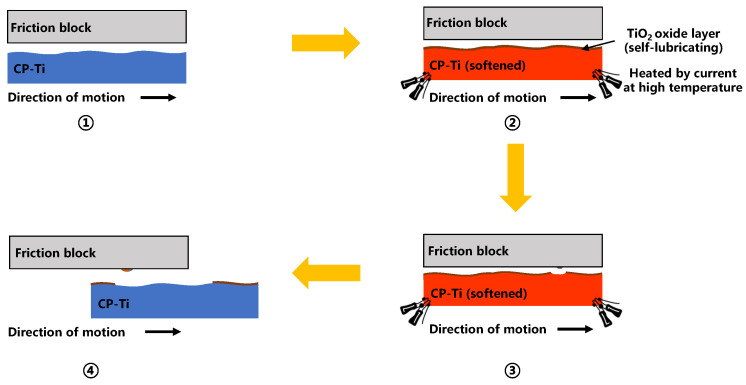
Mechanism of high-temperature friction in CP-Ti seet.

**Figure 16:**
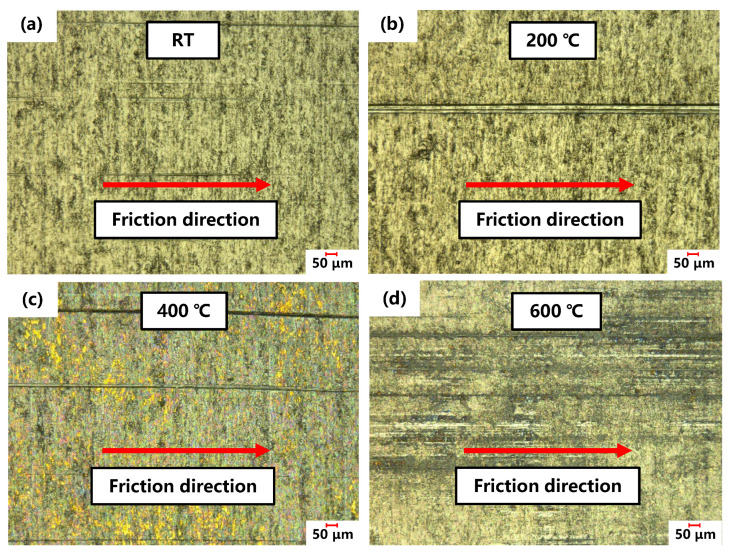
Micrographs of the scratches on the surface of the CP-Ti sheet at (**a**) RT, (**b**) 200, (**c**) 400, and (**d**) 600 °C.

Figure 15 illustrates the mechanism of high-temperature friction in a CP-Ti sheet, and Figure 16 shows images of the friction morphology of the CP-Ti sheet from RT to 600 °C. The scratches on the surface of the CP-Ti sheet at 25 °C were shallow and narrow, resulting in minimal hindrance to the mutual movement between the friction pairs and a small friction coefficient. However, the material softening effect at 200 °C led to asperities being embedded into the surface of the friction block. The abrasive particles generated during the friction process were embedded into the specimen surface, causing deeper scratches and a larger friction coefficient. As the temperature increased further, an oxide layer formed on the surface of the CP-Ti sheet, which current studies suggest may be TiO_2_. This TiO_2_ layer provided a certain degree of lubrication and reduced the friction coefficient [29,30]. Owing to the different material makeup of the CP-Ti sheet and friction block, atomic diffusion did not lead to material transfer, making the formation of the TiO_2_ a key factor that influenced the friction coefficient. The increase in friction temperature at 400 °C caused the formation of a significant TiO_2_ layer on the surface of the titanium sheet, which partially isolated the interaction between the friction blocks and the titanium base. Consequently, the surface scratches at this temperature were shallower and narrower than those observed at 200 °C, thereby reducing the friction coefficient. The surface morphology was distinctly different at 600 °C, where a thicker TiO_2_ oxide layer developed on the titanium surface. The friction morphology was primarily defined by the interaction between the oxide layer and the friction block, where the self-lubricating properties of the oxide layer led to a lower friction coefficient at 600 °C.

**Figure 17:**
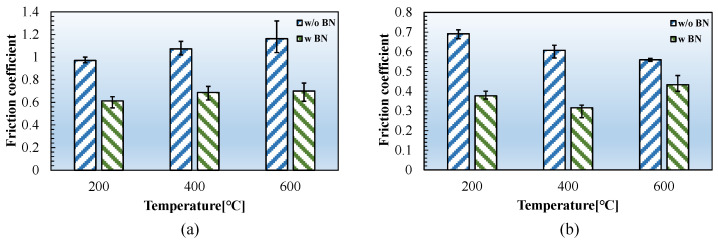
Friction coefficients at elevated temperatures with and without BN lubrication. (**a**) FSS and (**b**) CP-Ti.

The experimental findings reveal that the friction coefficients of FSS and CP-Ti are significantly higher at elevated temperatures than at RT. To address this issue, BN lubricant was applied to the specimen surface. The results obtained from friction experiments on FSS and CP-Ti at elevated temperatures, including BN lubrication, are presented in Figure 17. These results indicate that the friction coefficients of both materials consistently decrease after implementing BN lubrication. In the case of FSS, the most significant reduction occurred at 600 °C, when the friction coefficient decreased from 1.16–0.7. Similarly, the most significant reduction in the friction coefficient of CP-Ti was observed at 200 °C, with a decrease from 0.69–0.38. Thus, the experimental findings of this study suggest that BN lubrication significantly reduces the friction coefficient of ultrathin metal sheets at high temperatures, providing valuable insights to inform the hot-forming process.

## 4. Conclusions

In this study, a testing apparatus was developed to measure the high-temperature friction coefficients of ultrathin 0.1 mm thick metal sheets. The friction coefficients of FSS and CP-Ti were measured across temperatures ranging from RT to 600 °C. Furthermore, the variations in the friction coefficient of these materials at different temperatures were detailed, and their underlying mechanisms were analyzed, leading to the following conclusions:

1. Temperature significantly affected the friction coefficients of both the FSS and CP-Ti sheets. These coefficients were 0.19 and 0.18, respectively, at RT, and notably increased to 0.97 and 0.69, respectively, at 200 °C, corresponding to increases of 411 and 283%. The friction coefficient of the FSS sheet continuously increased with temperature, whereas that of the CP-Ti sheet slightly decreased with temperature.
2. The friction coefficient was significantly influenced by the atomic transfer between the friction pair and the oxide layer formed on the metal sheet surface. The FSS sheet experienced a higher rate of atomic transfer, owing to its compositional similarity with the friction block, leading to the formation of built-up edges and a significant increase in the friction coefficient.
3. The lower friction coefficient of the CP-Ti sheet at elevated temperatures, compared to that of the FSS sheet, was attributed to minimal atomic transfer and the formation of a self-lubricating TiO_2_ layer. Furthermore, the formation of a thicker oxide layer with increasing temperature led to a decrease in the friction coefficient of CP-Ti.
4. The application of BN lubricant notably reduced the friction coefficients of both FSS and CP-Ti ultrathin metal sheets at high temperatures.

## Figures and Tables

**Table 1:** Chemical composition of the 0.1 mm thick ferritic stainless steel (FSS) sheet.

Element	C	Cr	Ni	Mn	Mo	Co	Nb	Fe	Others
Comp. (wt. %)	0.02	27.32	1.84	0.19	3.64	0.02	0.35	66.28	Bal.

**Table 2:** Chemical composition of the 0.1 mm thick commercially pure titanium (CP-Ti).

Element	Fe	Mg	Na	Ni	Si	Ti	Others
Comp. (wt. %)	0.04	0.08	0.23	0.02	0.02	99.5	Bal.

## Data Availability

Data are contained within the article.
